# n‐3 polyunsaturated fatty acids improve depression‐like behavior by inhibiting hippocampal neuroinflammation in mice via reducing TLR4 expression

**DOI:** 10.1002/iid3.707

**Published:** 2022-10-11

**Authors:** Li Hu, Xianxiang Zeng, Kai Yang, Hongli Peng, Jinhong Chen

**Affiliations:** ^1^ Department of Sleep Disorders and Neuroses Brain Hospital of Hunan Province Changsha Hunan Province China; ^2^ Department of Clinlical Psychology Brain Hospital of Hunan Province Changsha Hunan Province China

**Keywords:** depression, inflammation, n‐3 PUFAs, TLR4

## Abstract

**Introduction:**

n‐3 polyunsaturated fatty acids (PUFAs) are believed to be implicated in the pathogenesis of many inflammation‐related diseases, including depression.

**Methods:**

The mouse model of depression was established through chronic unpredictable mild stress (CUMS), the mice were intervened with n‐3 PUFAs, and then the expression of toll‐like receptor 4 (TLR4) was stimulated with lipopolysaccharides (LPS). Tail suspension test (TST), forced swimming test (FST) and sucrose preference test were performed to monitor the depression behavior of mice. Microglia activation was detected by Iba1 immunofluorescence, and neuronal injury was detected by Nissl staining. Concentrations of tumor necrosis factor (TNF)‐α, Interleukin (IL)‐6 and IL‐1β in the hippocampus were assessed via enzyme linked immunosorbent assay (ELISA). Quantitative real time polymerase chain reaction was used to detect IL‐6, IL‐1β and TNF‐α messenger RNA levels. Western blot was utilized for detection of TLR4 protein expression.

**Results:**

CUMS significantly reduced the sucrose preference in mice, while increased the immobility time in FST and TST. Moreover, CUMS significantly aggravated microglia activation and neuronal damage in mice and increased the levels of IL‐6, IL‐1β and TNF‐α in hippocampal tissues, however, intervention with n‐3 PUFAs could improve the above effects. Further, the increased TLR4 induced by LPS partially reversed the inhibition of n‐3 PUFAs on depression‐like behaviors, microglial activation and inflammatory injury of hippocampal neurons.

**Conclusion:**

n‐3 PUFAs may ameliorate depression‐like behaviors via reducing hippocampal neuroinflammation in CUMS‐induced mice by regulating TLR4 expression, suggesting that n‐3 PUFAs may be an effective antidepressant, which provides evidence for future treatment of depression.

## Graphical abstract

n‐3 polyunsaturated fatty acids (PUFAs) are believed to be implicated in the pathogenesis of many inflammation‐related diseases, including depression. n‐3 PUFAs may ameliorate depression‐like behaviors via reducing hippocampal neuroinflammation in chronic unpredictable mild stress‐induced mice by regulating toll‐like receptor 4 expression, suggesting that n‐3 PUFAs may be an effective antidepressant, which provides evidence for future treatment of depression.

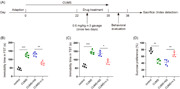

## 1 INTRODUCTION

Depressive disorder, a common emotional disorder or affective disorder, appears with a main clinical feature of obvious and lasting depression that easily recurs. In severe cases, it could even lead to suicidal behaviors.^1^ The pathogenesis of depression is complex, involving multisystem and multilink dysfunction. And its incidence is mostly believed to be caused by the interaction of social, environmental and individual factors.^2^, ^3^ At present, the pathogenesis of depression mainly includes the imbalance of monoamine neurotransmitters, neurogenesis disorders, oxidative stress disorders, and immune regulation disorders. Although there are many etiological hypotheses, there is still no definitive conclusion.^4^

Polyunsaturated fatty acids (PUFA) can be divided into n‐3 PUFAs and n‐6 PUFAs according to the position of the first double bond from the methyl end.^5^ Among which, n‐3 PUFAs mainly includes docosahexaenoic acid (DHA) and eicosapentaenoic acid (EPA),^6^ which are important and indispensable nutrients that human body cannot synthesize and thus called essential fatty acids.^7^ Studies have shown that the content of n‐3 PUFAs in the peripheral serum and red blood cell membrane of patients with depression was significantly reduced.^8^ Edwards et al.^9^ also found that n‐3 PUFAs could inhibit the levels of phospholipids and cholesterol lipids in patients with depression, and the degree of decrease was related to the severity of depression. However, the functional roles and mechanisms of n‐3 PUFAs in depression still remain elusive. In addition, there are clinical studies showing that n‐3 PUFAs can play a therapeutic role in people at high risk of psychosis. Since n‐3 PUFAs supplements can be easily and safely used in a variety of settings from primary care to specialty services,^10^ further investigation of the n‐3 PUFAs relationship with depression is warranted, which may help to help people with depression.

Toll‐like receptor (TLR) is a type I transmembrane protein, composed of extracellular leucine‐rich repeat sequence domain, intracellular conserved Toll/Interleukin (IL)‐1 receptor domain and transmembrane domain.^11^ TLRs are the body's first line of immune defense against the invasion of external microorganisms, as well as a bridge connecting innate immunity and adaptive immunity, and belong to the pattern recognition receptor family.^12^ TLR4, a member of the TLR family, has been found in a growing number of studies that inhibition of TLR4 or related signaling pathways could improve depression even the occurrence of depression.^13^ For example, lipopolysaccharides (LPS) or heat shock proteins or TLR4 endogenous ligands high‐mobility‐group box 1 could trigger the innate immune system via TLR4 signaling pathway and induce pathological behaviors among animal models which are similar to the depressive symptoms.^14^, ^15^ In addition, a large amount of dietary n‐3/n‐6 PUFAs ameliorated obesity‐related insulin resistance and inflammation by inhibiting the activation of TLR4 in rats,^16^ prompting us to speculate whether n‐3 PUFAs could suppress depression by regulating TLR4 and its related signaling pathways.

In this article, we considered and explored the effects of n‐3 PUFAs on depression‐like behavior and hippocampal neuroinflammation in chronic unpredictable mild stress (CUMS) induced mice. Moreover, the anti‐depressant activity of n‐3 PUFAs is mediated by inhibition of TLR4, suggesting n‐3 PUFAs or TLR4 control may be a therapeutic strategy for depression.

## 2 MATERIALS AND METHODS

### 2.1 Animals and experimental protocol

42 C57BL/6 mice (male, weighted 18−22 g, and aged 8 weeks) in this study were provided by Hunan Slack Jingda Experimental Animal Co., Ltd. Each group of six mice was raised in separate cages with temperature adjusted to 23−25°C and relative humidity to 40%−60%. All mice could eat freely and were raised with national standard rodent feed. CUMS was applied to construct a mouse model of depression and mice were first separated into following groups: CUMS group, CUMS + n‐3 PUFAs group, CUMS + normal saline (NS) group and control group. Following stressors were used to induce mice in CUMS group^1^: water deprivation (24 h),^2^ fasting (24 h),^3^ overnight illumination,^4^ cage tilting (45°),^5^ wet sawdust (100 g sawdust padding with 200 ml water),^6^ contact with foreign matter,^7^ reversal of light and dark cycles,^8^ overhang (10 min),^9^ exposure to an empty bottle,^10^ tail pinch (1 min, 1 cm from the root of the tail),^11^ shaking (5 min), and^12^ white noise. Steps mentioned above were randomly arranged to guarantee the unpredictability of the test and lasted for 5 weeks. The control group was placed in a separate room under the same conditions, without contact with the stressed animals. Starting from Day 22, mice in the CUMS + NS group were given 0.9% saline by gavage, while DHA (Sigma) was given by gavage to the mice in CUMS + n‐3 PUFAs group once every 2 days at a dose of 0.6 mg/kg. The dosing time was simply modified with reference to the previous literature.^17^ Mice were euthanized after the forced swimming test (FST) and with their hippocampus collected. In addition, after constructing CUMS, LPS was used to specifically activate TLR4 in mice,^18^ and CUMS mice were divided into CUMS + n‐3 PUFAs group, CUMS + n‐3 PUFAs + phosphate buffer saline (PBS) group and CUMS + n‐3 PUFAs + LPS group. 0.83 mg/kg of LPS (Sigma) in PBS solution was injected into CUMS+ n‐3 PUFAs + LPS group by intraperitoneal injection 1 h before n‐3 PUFAs gavage, and an equal volume of PBS were injected into the mice in CUMS + n‐3 PUFAs + PBS group. After behavioral tests, mice were killed and their brain tissues were collected for subsequent procedure. All animal experiments had gained approval from the Medical Ethics Committee of Brain Hospital of Hunan Province (Approval No.: 2020137) and were carried out in accordance with the Guidelines for the Care and Use of Animals for Scientific Research.

### 2.2 Sucrose preference test (SPT)

Before test, the mice were given sugar water training to measure the baseline level of the syrup bias of each group of mice. After fasting for 24 h, the mice were given two bottles of preloaded and quantified water, one bottle of tap water and another containing 2% sucrose solution. After 12 h of free drinking, the bottle was weighted for calculation of consumption. Sucrose preference was analyzed with the formula as follow: (sucrose preference) = ([sucrose intake]/[sucrose intake + water intake]) × 100.

### 2.3 FST

The mouse was placed alone in a glass cylinder (diameter 20 cm, height 40 cm) containing 25 cm deep water under constant temperature at about 28°C and forced mouse to pre‐swim for 15 min. After 24 h, the mouse was forced to swim again under the same experimental conditions, and the immobile time of mouse in the tank within 5 min was recorded. Immobility referred to the time required for a mouse to float in the water without struggling or just keeping its head above the water surface.

### 2.4 Tail suspension test (TST)

The mice were individually suspended by a tape (2 cm from the end) on the tail 15 cm above the floor. Each mouse was recorded by a video camera with their immobility time calculated in the last 4 min during the 6‐min test and reported in seconds. Only when the mouse was suspended passively and stayed stationary completely could it be defined as immobile.

### 2.5 ELISA

After the mice were anesthetized, the mice were killed by cervical dislocation, and their hippocampal tissues were subsequently dissected and frozen in liquid nitrogen. The hippocampus tissue was weighed and cut into pieces (1−3 mm) and subsequently prepared into a 10% homogenate with an ultrasonic cell crusher (Shanghai Billion Instrument Co., Ltd.). After centrifugation for 15 min at 4°C, supernatants were taken for testing the levels of IL‐1β (Abcam, ab214025), tumor necrosis factor (TNF)‐α (Abcam, ab181421), and IL‐6 (Abcam, ab178013) with corresponding ELISA kits.

### 2.6 Quantitative real time polymerase chain reaction (qPCR) detection

Relative messenger RNA (mRNA) levels of TNF‐α, IL‐1β and IL‐6 were assessed via qPCR assay. Briefly, Trizol reagent (Invitrogen) was applied for extraction of total RNA in hippocampal tissue fragments, which was reverse‐transcribed into complementary DNA (cDNA) by a cDNA Synthesis Kit (Takara). After transcription, 1 μl of synthesized cDNA was taken for qPCR using SYBR‐Green reagent kits (Takara). β‐actin were used as internal control and sequences of primers were as follows:

IL‐6 (forward): 5′‐TGCAAGAGACTTCCATCCAG‐3′,

IL‐6 (reverse): 5′‐TCCACGATTTCCCAGAGAAC‐3′;

IL‐1β (forward): 5′‐GCCACCTTTTGACAGTGATGAG‐3′,

IL‐1β (reverse): 5′‐AAGGTCCACGGGAAAGACAC‐3′;

TNF‐α (forward): 5′‐CCGATGGGTTGTACCTTGTC‐3′,

TNF‐α (reverse): 5′‐TGGAAGACTCCTCCCAGGTA‐3′;

β‐actin (forward): 5′‐CCCCTGAACCCTAAGGCCA‐3′,

β‐actin (reverse): 5′‐CGGAGTCCATCACAATGCCT‐3′.

### 2.7 Nissl staining

The hippocampal tissue sections were deparaffinized and hydrated, rinsed with distilled water for three times (5 min each), placed in cresyl violet staining solution, soaked at 56°C for 1 h, and then rinsed with deionized water. Next, sections were placed in Nissl differentiation solution (Solarbio) for 1−2 min, and continue to be dehydrated and fixed with natural resin. Finally, Nissl bodies in neuronal cytoplasm and dendrites were observed under a light microscope.

### 2.8 Immunofluorescence

Immunohistochemistry was performed to estimate the proportion of Iba1^+^ microglia in the brain tissue of mice. The paraffin‐embedded brain tissue was cut into 5 µm‐thick sections using a microtome. After deparaffinization and rehydration, sections were treated with fresh 3% H_2_O_2_ for 25 min at room temperature to eliminate endogenous peroxidase activity. After several washes with PBS, sections were blocked with 3% bovine serum albumin for 30 min and then with primary antibody Iba1 (Abcam; ab178846; 1:2000) overnight at 4°C. Subsequently, further incubation with HRP‐IgG was conducted for 30 min at room temperature. Washed with PBS again, sections were then stained with diaminobenzidine and counterstained with hematoxylin, and were observed with light microscope (Nikon). Five different fields were selected in each section to count Iba1 positive cells.

### 2.9 Western blot

RIPA buffer (Beyotime) was utilized for extraction of total protein in brain hippocampal tissues from each group, and phosphatase and protease inhibitors were then added. After grinding with liquid nitrogen and centrifuging at 12,000 rpm under 4°C for 30 min, we collected and stored the supernatant protein in a refrigerator at −80°C. The concentration of the sample was determined with the BCA kit (KeyGen) and was uniformly 2 μg/μl. The protein lysate was parted by 10% sodium dodecyl sulfate polyacrylamide gel electrophoresis (SDS‐PAGE) and transferred to a polyvinylidene fluoride membrane. After blocking with 5% BSA for 1 h, the following antibodies were used: TLR4 (Abcam; ab13556; 1:1000) and β‐actin (Abcam; ab8227; 1:2000). After incubation with primary antibodies overnight at 4°C, membrane was then nurtured with horseradish peroxidase‐conjugated secondary antibody (Abcam; ab205718; 1:10000) for 1 h. And chemiluminescence imaging system (Bio‐Rad) was applied to visualize protein bands.

### 2.10 Statistical analysis

All data were shown as means ± standard deviations. Statistical analyses were conducted using SPSS18.0 software, and pictures required were drawn with GraphPad Prism 8 software. Differences between multiple groups were analyzed using One‐way analysis of variance with Tukey's test. When *p* < .05, it was considered statistically significant.

## 3 RESULTS

### 3.1 n‐3 PUFAs ameliorated depression‐related behaviors among mice induced by CUMS

**Figure 1:**
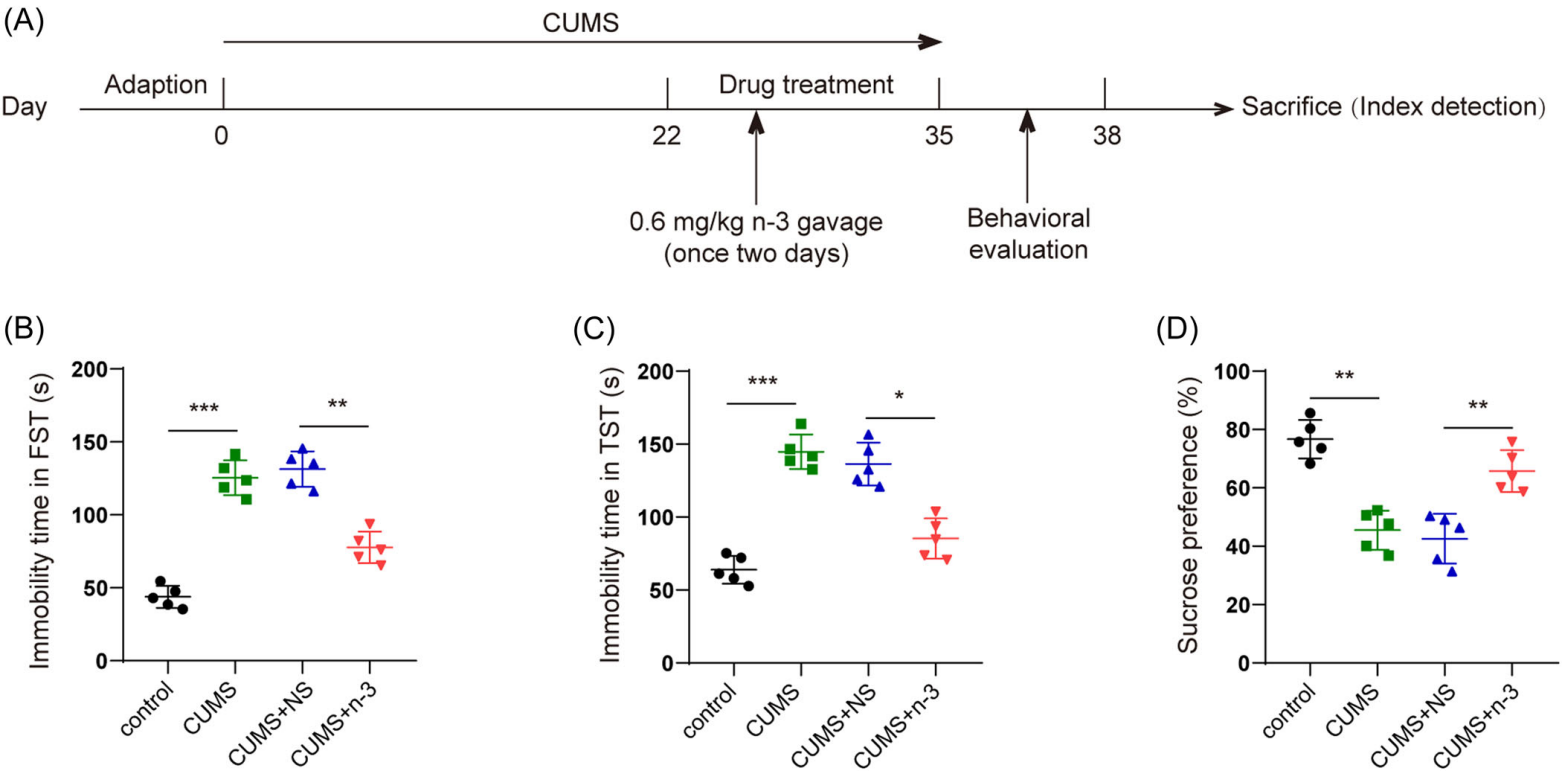
n‐3 PUFAs improved the depressive behavior of mice induced by CUMS. (A) Experimental schedule of mouse treatment. (B, C) TST, FST evaluation of the immobility time of CUMS‐induced mice after n‐3 PUFAs intervention. (D) SPT evaluation of the sucrose preference of CUMS mice after n‐3 PUFAs intervention. *p < .05, **p < .01, ***p < .001.CUMS, chronic unpredictable mild stress; FST, forced swimming test; PUFAs, polyunsaturated fatty acids; SPT, sucrose preference test; TST, tail suspension test.

We set up a mouse model undergoing depression through CUMS induction and subsequently conducted TST, FST, and SPT tests (Figure 1A). The immobility time of FST and TST were significantly increased by CUMS treatment, while n‐3 PUFAs notably declined the immobility time of mice (Figure 1B, C). Under the same reference, SPT results showed that the sucrose preference rate of mice was strikingly declined in CUMS group, which was partially reversed by n‐3 PUFAs (Figure 1D). Results mentioned above suggested that n‐3 PUFAs could inhibit CUMS‐induced depression behavior in mice.

### 3.2 n‐3 PUFAs suppressed hippocampal neuroinflammation in mice induced by CUMS

**Figure 2:**
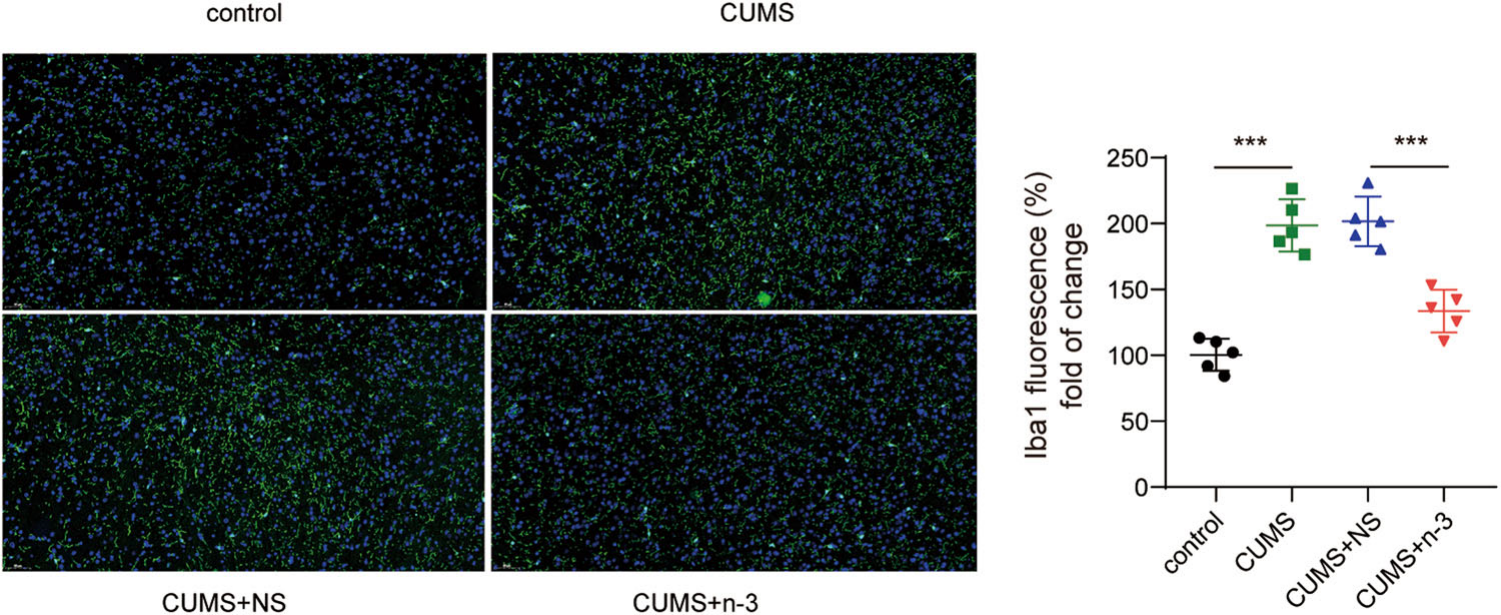
n‐3 PUFAs reduced the activation of microglia in mice induced by CUMS. Immunofluorescence detection of microglia activation. ***p < .001. CUMS, chronic unpredictable mild stress; PUFAs, Polyunsaturated fatty acids.

**Figure 3:**
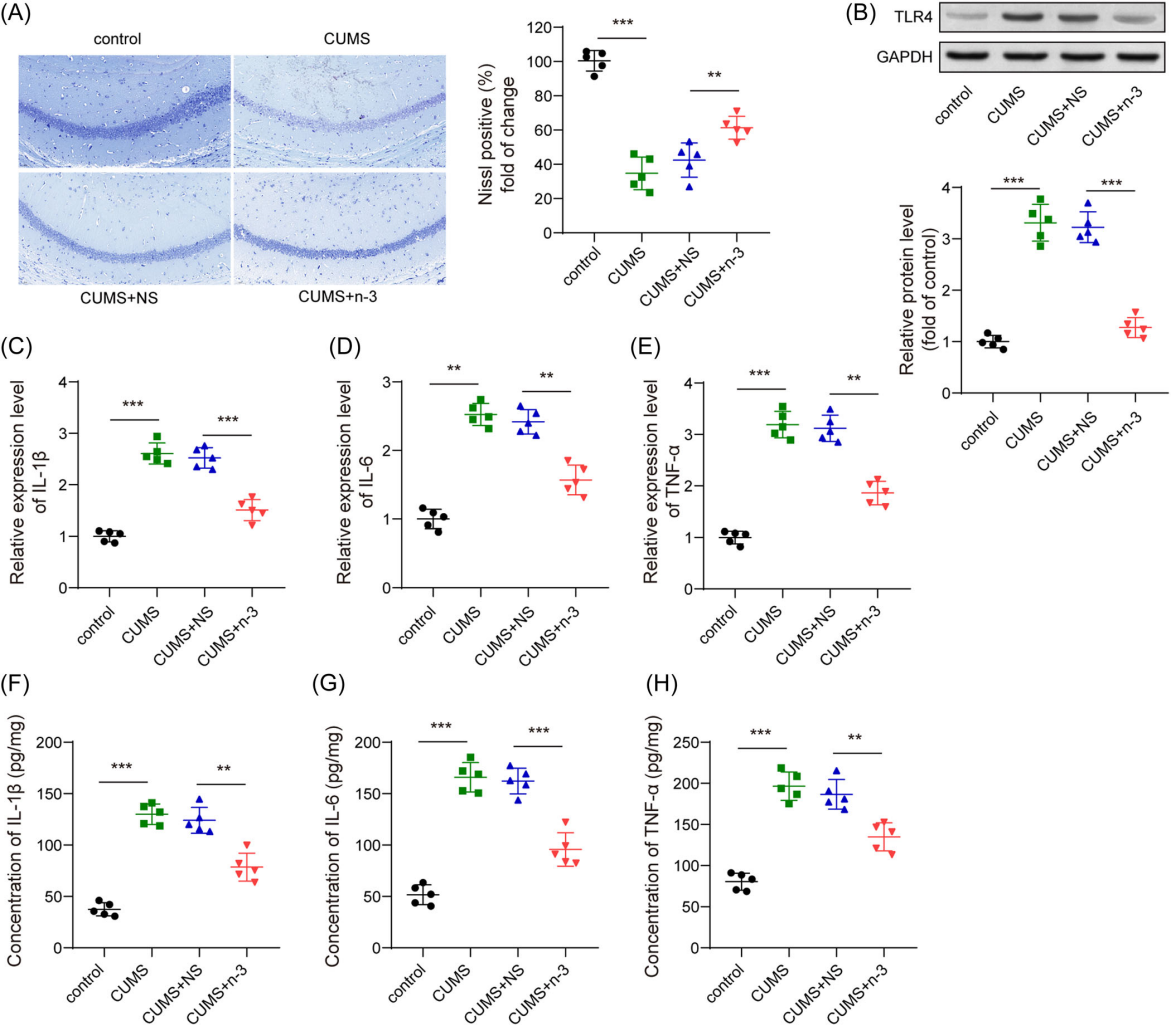
n‐3 PUFAs suppressed hippocampal neuroinflammation in mice induced by CUMS. (A) Nissl staining detection of neuron damage. (B) Western blot detection of the expression of TLR4 protein. (C−E) qPCR detection of IL‐6 mRNA, IL‐1β mRNA and TNF‐α mRNA in the hippocampus of mice. (F−H) ELISA measurement of the concentration of inflammatory factors IL‐6, IL‐1β, and TNF‐α in the hippocampus of mice. **p < .01, ***p < .001. CUMS, chronic unpredictable mild stress; IL, Interleukin; mRNA, messenger RNA; PUFAs, polyunsaturated fatty acids; qPCR,Quantitative real time polymerase chain reaction; TLR4, toll‐like receptor 4; TNF, tumor necrosis factor.

It was found by Iba1 fluorescence staining that compared with the control group, the microglia in the CUMS group were significantly activated, while the activation of the microglia in the CUMS + n‐3 PUFAs group was evidently inhibited (Figure 2). Moreover, Nissl staining indicated that n‐3 PUFAs could improve CUMS‐caused hippocampus neuronal injury (Figure 3A). Then, we determined hippocampal neuroinflammation in depression mice. Compared with the control group, CUMS had greatly risen the TLR4 protein expression in the hippocampus of mice, while n‐3 PUFAs repressed TLR4 level upregulated by CUMS (Figure 3B). qPCR test found that the expression of IL‐1β, IL‐6 and TNF‐α mRNA in mouse hippocampus were promoted by CUMS, but n‐3 PUFAs exhibited a suppressive effect on the expression of inflammatory cytokines (Figure 3C−E). Furthermore, ELISA assay also found that n‐3 PUFAs reduced the concentrations of IL‐1β, TNF‐α and IL‐6 in mouse hippocampus induced by CUMS treatment (Figure 3F−H). Collectively, n‐3 PUFAs could reduce the inflammatory damage of the hippocampal neurons in mice induced by CUMS, which may be related to TLR4 inhibition.

### 3.3 Activation of TLR4 partly reversed the inhibitory effect of n‐3 PUFAs on depression‐like behavior in mice

**Figure 4:**
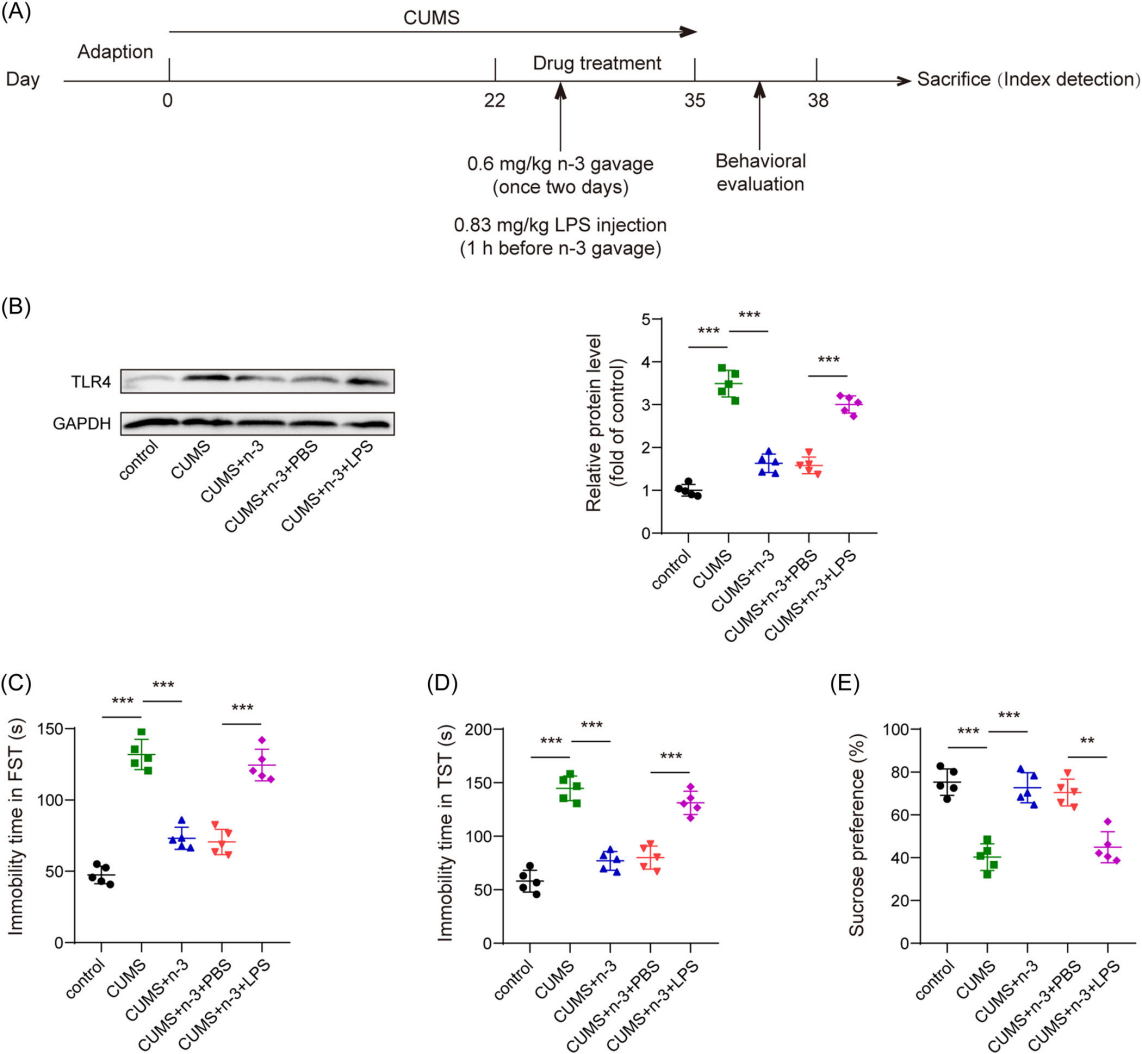
TLR4 activation could reverse the inhibitory effect of n‐3 PUFAs on depression‐like behavior among mice. (A) Experimental schedule of mouse treatment. (B) Western blot detection of the expression of TLR4 protein in the hippocampus among mice. (C, D) TST and FST evaluation of the immobility time of mice. (E) SPT assessment of the sucrose preference of mice. **p < .01, ***p < .001. FST, forced swimming test; PUFAs, polyunsaturated fatty acids; SPT, sucrose preference test; TLR4, toll‐like receptor 4; TST, tail suspension test.

Next, we activated TLR4 utilizing LPS stimulation to explore whether the effect of n‐3 PUFAs depended on the repression of TLR4(Figure 4A). First, LPS upregulated the protein level of TLR4 in hippocampus from n‐3 PUFAs treated depression mice (Figure 4B). Subsequently, the results of motivational behaviors in n‐3 PUFAs and CUMS induced mice showed that overexpression of TLR4 evidently rose immobility time in FST and TST, while declined sucrose preference in SPT (Figure 4C‐E). Taken together, TLR4 activation could reverse the inhibitory effect of n‐3 PUFAs on depression‐like behavior of the mice.

### 3.4 TLR4 activation partially reversed the inhibition of n‐3 PUFAs on hippocampal neuroinflammation

**Figure 5:**
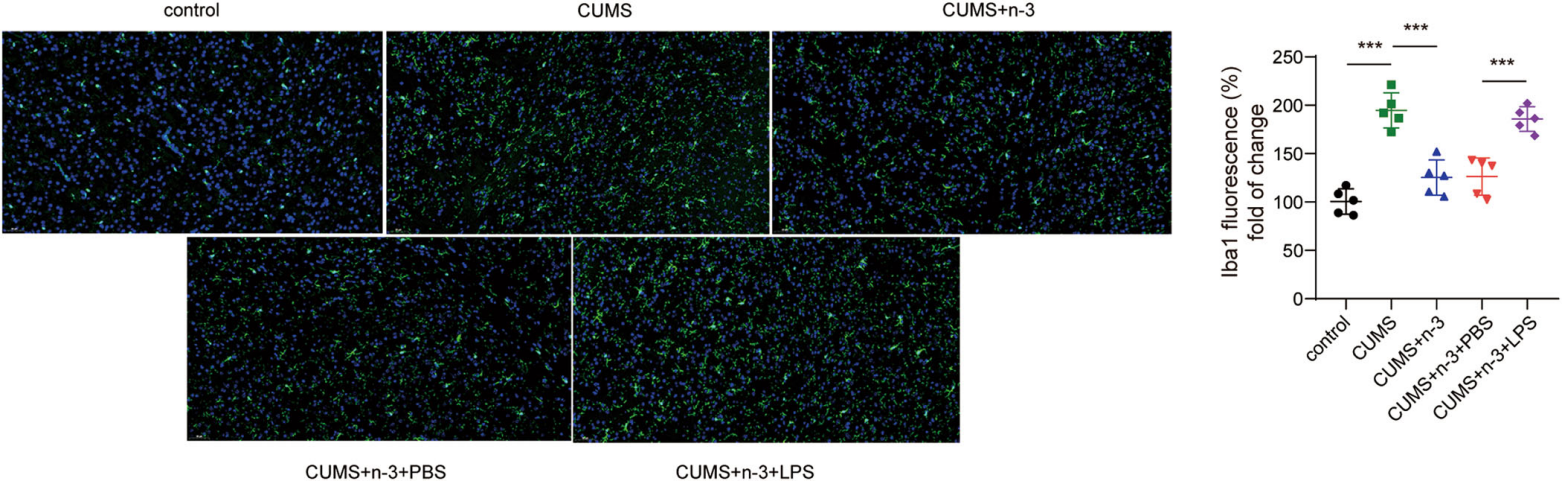
TLR4 activation could reverse the inhibitory effect of n‐3 PUFAs on microglia activation. Immunofluorescence detection of microglia activation. ***p < .001. PUFAs, polyunsaturated fatty acids; TLR4, toll‐like receptor 4.

**Figure 6:**
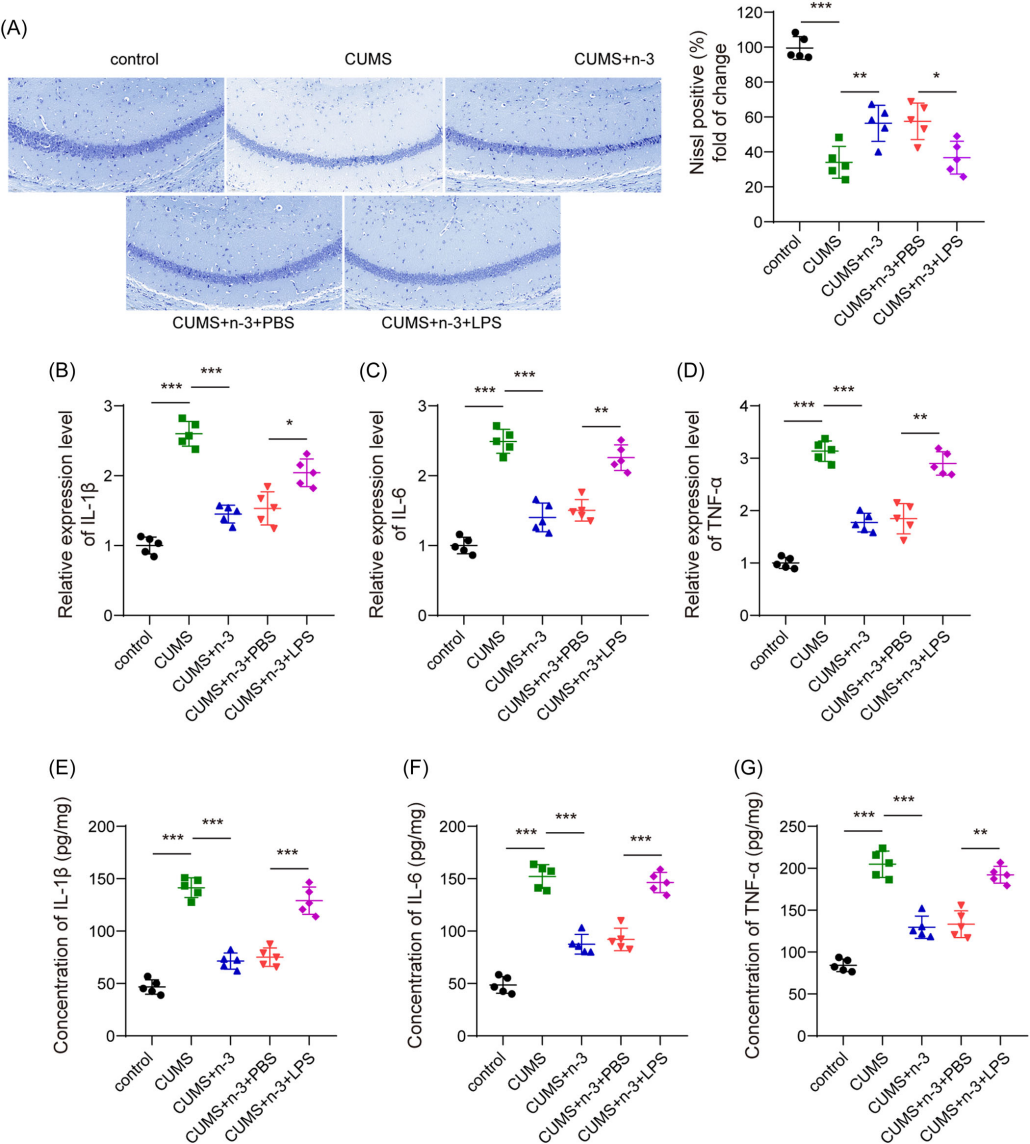
TLR4 activation could reverse the inhibitory effect of n‐3 PUFAs on hippocampal neuroinflammation. (A) Nissl staining detection of neuron damage. (B−D) qPCR detection of IL‐6 mRNA, IL‐1β mRNA and TNF‐α mRNA in the hippocampus of mice. (E‐G) ELISA detection of the concentration of IL‐6, IL‐1β, and TNF‐α of hippocampus in mice. *p < .05, **p < .01, ***p < .001. IL, Interleukin; mRNA, messenger RNA; PUFAs, polyunsaturated fatty acids; qPCR,Quantitative real time polymerase chain reaction; TLR4, toll‐like receptor 4; TNF, tumor necrosis factor.

Iba1 fluorescence staining showed that LPS‐induced TLR4 activation partially reversed the activation of microglia decreased by n‐3 PUFAs in depression mice (Figure 5). Furthermore, TLR4 activation promoted the n‐3 PUFAs alleviated neuronal damage in CUMS‐induced mice (Figure 6A). Then, results from qPCR showed that the expression of IL‐6, IL‐1β and TNF‐α mRNA in the hippocampal tissues of the CUMS + n‐3 PUFAs + LPS treated mice was significantly increased (Figure 6B−D). Consistently, the concentrations of IL‐6, IL‐1β, and TNF‐α in the hippocampus of mice in CUMS + n‐3 PUFAs + LPS group also elevated strikingly (Figure 6E–G), suggesting that TLR4 activation could reverse the inhibition of n‐3 PUFAs on the release of inflammatory factors in hippocampus of mice.

## 4 DISCUSSION

With the accelerated pace of life in modern society, depression has become one of the most common mental illnesses that has lifelong impact on patients.^19^ It is an inflammatory disease closely related to the regulation of emotions and mood disorders in the hippocampus of human brain, and is manifested as long‐term depression and typical “three highs and three lows” (high prevalence, high disability, high recurrence, and low detection rate, low consultation rate, low cure rate). Severe cases of depression can lead to suicide or extended suicide, which has become the main killer of mankind in the 21st century and the most serious health problem plagued the world.^20^ The mammalian brain and central nervous system are especially dependent on the n‐3 fatty acid DHA for normative signaling and function, and DHA deficiencies is one contributing factor in the increasing prevalence of depressive disorders.^21^ Moreover, DHA may help prevent and treat depression by virtue of their effects on neurogenesis in the hippocampus.^21^ In this study, DHA was used to supplement n‐3 PUFAs and it was observed that n‐3 PUFAs could improve the depression‐like behavior and inflammatory response in depression mice by inhibiting TLR4 expression.

More and more epidemiological and dietary studies have shown that most depressed patients are deficient in n‐3 PUFAs, and supplementation of which can improve depressive symptoms and behavior.^22^ n‐3 PUFAs are essential fatty acids of important biological significance, which cannot be synthesized in the human body spontaneously and must be obtained from food and daily diet. Its dynamic balance plays an important role in the stability of human body's environment and normal growth and development, which is mainly reflected in stabilizing cell membrane structure, regulating gene expression, and maintaining the balance of cytokines and lipoproteins.^23^, ^24^ The intake of n‐3 PUFAs can be reflected in a variety of red blood cells and plasma lipids. Animal models indicated that the PUFA content of red blood cells could be taken as an indicator of the composition of brain cell membranes.^25^ Studies have revealed that increasing n‐3 PUFAs intake could exert inhibitory effect on major depressive disorder in adults.^26^ In present study, n‐3 PUFAs was used to intervene in CUMS‐induced depression mice to verify that n‐3 PUFAs could significantly improve depression‐related behaviors, inflammation in hippocampus, neuronal damage, and microglia activation. With the development of research on the connection between depression and n‐3 PUFAs, more and more animal studies aim at exploring the mechanisms of n‐3 PUFAs working on depression. Research have shown that long‐term supplementation of fish oil (rich in DHA and EPA) in female rats before and after parturition could reduce behavioral disturbances in their offspring in the Modified Forced Swim Test (swimming, climbing, and immobility), suggesting that long‐term supplementation of n‐ 3 PUFAs may be effective in depression treatment.^27^ In addition, long‐term n‐3 PUFAs intake could trigger antidepressant‐like efficacy and inhibit the expression of IL‐6,^28^ which is consistent with our observations. Although a number of studies have shown that there was a negative relationship between depression and n‐3 PUFAs, further study is required to clarify the mechanism of n‐3 PUFAs working on depression. Previous studies have suggested that the effect of n‐3 PUFAs on depression was related to its metabolic mechanism. It is believed that n‐3 PUFAs are metabolized by soluble epoxide hydrolase, and soluble epoxide hydrolase plays a key role in inflammation, so that it may also play a role in the pathogenesis of mental illness.^29^, ^30^

However, the pathogenesis of depression is complex, which includes a variety of molecular mechanisms and signaling pathways, studies have found that TLR4 signaling pathway was activated in the peripheral circulatory system or central nervous system (CNS) of depression patients and depression animal models.^31^ TLR4 is mainly expressed in brain microglia, and adult nerves in the subgranular region of the hippocampus dentate gyrus were involved in the process of learning, memory and emotional management.^32^ Previous studies have found that microglia could ameliorate depression by modulating neuroinflammation through M1 and M2 subtypes.^33^ Another study found that activation of microglia and neuro‐improvement in the adult hippocampus reduced depression‐like behaviors in mice.^34^ This is further evidence that there is a strong link between neuroinflammation and depression. Another study found that TLR4 activation in the rat hippocampus could induce microglial activation and neuroinflammation, which more directly reflects the relationship between TLR4 and the hippocampus.^35^ Previous studies have found that safflower extract could improve depression in mice by inhibiting TLR4/NLRP3 inflammatory signaling pathway.^36^ Another study found that inhibition of TLR4 expression could reduce neuroinflammation‐induced depression‐like behavior.^37^ Therefore, the activation of TLR4 in the CNS may be implicated in the pathophysiological process of some depressive symptoms. Additionally, it was found that LPS could set off the innate immune system via TLR4 signaling pathway and induce pathological behaviors among animal models, which is similar to the symptoms of depression among humankind in animal experiments.^14^ In our study, TLR4 expression in the hippocampus of mice w also stimulated by LPS, and it was found that TLR4 can reverse the inhibition of n‐3 PUFAs on the depressive behavior, inflammatory response, neuronal damage, and microglia activation of depression mice, which suggests that depression‐like behavior and hippocampal neuroinflammation in mice may be improved by n‐3 PUFAs through suppressing TLR4 pathway. The terminal of the TLR4 signaling pathway is the release of various cytokines, and peripheral cytokines can transmit signals to the brain through molecular, cellular and neurotransmission pathways, thereby causing or promoting CNS inflammation.^38^ Both acute and chronic stress could activate the peripheral or central TLR4 signaling pathway in rodents and induce depression‐like behaviors.^39^, ^40^ Moreover, TLR4 was able to regulate the response of adrenal hormones to stress and inflammatory stimuli, and NF‐κB could disrupt hypothalamic−pituitary−adrenal axis to cause inflammatory response disorder, thereby aggravating the symptoms of depression.^41^ In our study, it was found that n‐3 PUFAs was able to improve depression by regulating TLR4.

In summary, we found that n‐3 PUFAs was able to improve the depressive behavior and hippocampal neuroinflammation in mice with depression, the mechanism of which may be achieved by regulating TLR4 pathway. Therefore, n‐3 PUFAs may be an efficient substance for treating depression, but it still needs to be confirmed by further studies.

## AUTHOR CONTRIBUTIONS

**Li Hu**: Concepts, design, funding acquisition, writing − reviewing and editing. **Xianxiang Zeng**: Data acquisition, data analysis. **Kai Yang**: Experimental studies. **Hongli Peng**: Data acquisition, data analysis. **Jinhong Chen**: Concepts, design, supervision, writing − original draft preparation. All the authors approved for the final version.

## CONFLICT OF INTEREST

The authors declare no conflict of interest.

## ETHICS STATEMENT

All animal experiments had gained approval from the Medical Ethics Committee of Brain Hospital of Hunan Province and were carried out in accordance with the Guidelines for the Care and Use of Animals for Scientific Research

## Data Availability

The data sets used or analyzed during the current study are available from the corresponding author on reasonable request.
